# Lags and leads of accommodation in humans: Fact or fiction?

**DOI:** 10.1167/jov.21.3.21

**Published:** 2021-03-25

**Authors:** Vivek Labhishetty, Steven A. Cholewiak, Austin Roorda, Martin S. Banks

**Affiliations:** 1 Optometry & Vision Science, University of California, Berkeley, CA, USA

**Keywords:** accommodation, visual acuity, wavefront aberrations, myopia, head-mounted displays

## Abstract

The focusing response of the human eye — accommodation — exhibits errors known as lags and leads. Lags occur when the stimulus is near and the eye appears to focus farther than the stimulus. Leads occur with far stimuli where the eye appears to focus nearer than the stimulus. We used objective and subjective measures simultaneously to determine where the eye is best focused. The objective measures were made with a wavefront sensor and an autorefractor, both of which analyze light reflected from the retina. These measures exhibited typical accommodative errors, mostly lags. The subjective measure was visual acuity, which of course depends not only on the eye's optics but also on photoreception and neural processing of the retinal image. The subjective measure revealed much smaller errors. Acuity was maximized at or very close to the distance of the accommodative stimulus. Thus, accommodation is accurate in terms of maximizing visual performance.

## Introduction

**Figure 1.**
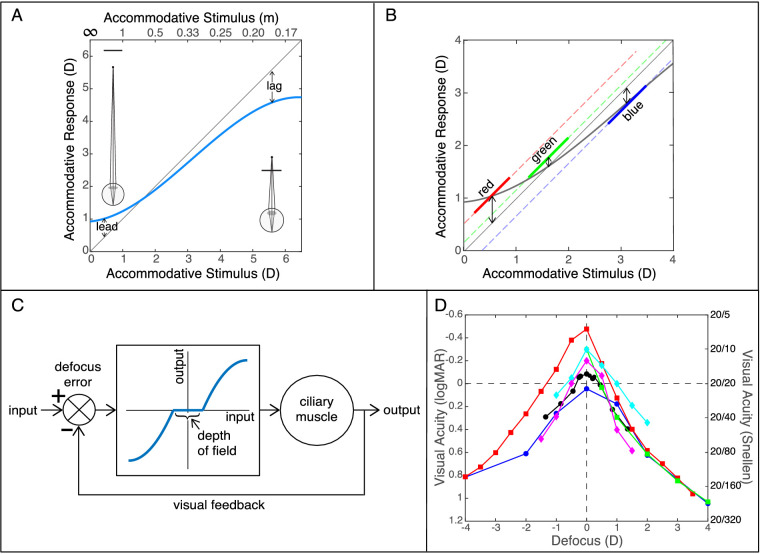
Stimulus–response curve, chromatic aberration, accommodation control system, and visual acuity. (A) Accommodative stimulus–response curve. Accommodative response in diopters is plotted against stimulus distance in diopters. For reference, the distances in meters are shown on top. The gray diagonal line is where accommodative response would precisely match the accommodative stimulus. The blue curve represents commonly reported data. It exhibits errors relative to the ideal response: lags at large diopter values (near distances) and leads at small diopter values (far distances). An accommodative lag is schematized on the right where the stimulus (black line) is near and the eye has focused farther than the stimulus. A lead is schematized on the left where the stimulus is far and the eye has focused nearer than that. (B) Chromatic aberration theory. This theory of lags and leads states that the eye, when presented a polychromatic stimulus, strategically focuses the longer wavelengths in that stimulus when it is far (left side of graph), middle wavelengths when it is at medium distance (middle), and short wavelengths when the stimulus is near (right). (C) Control system model of accommodation. The input is the desired power of the crystalline lens: that is, the value needed for optimal focus at the retina. The output is the actual lens power. Actual value is subtracted from desired at the comparator. The controller (central box) converts the output of the comparator into a neural signal to drive the ciliary muscle and thereby change lens power. The controller has a “dead zone” around zero where blur is not perceptible due to the eye's depth of focus. The falling and rising parts of the input–output curve represent errors that drive the ciliary muscle's action in the correct direction to minimize defocus. The falling and rising parts decrease slope at the extremes to yield the farthest and nearest distances to which the lens can adjust state. (D) Visual acuity as a function of defocus. Letter acuity (logMAR on the left, Snellen on the right) is plotted against the sign and magnitude of defocus in diopters. A logMAR acuity of 0 (horizontal dashed line) is 20/20, where the strokes of the just-identifiable letters subtend 1minarc. Better acuities are upward. The red squares are from Tucker and Charman (1975) (5-mm pupil, subject WNC). The cyan diamonds are from Zheleznyak et al. (2013) (5 mm, dominant eye; defocus values adjusted to compensate for spectacle lens power). The magenta diamonds are from Legras et al. (2010) (4 mm, average of four subjects). The black circles are from Guo et al. (2008) (5.5 mm, average of two subjects). The blue circles are from Legge et al. (1987) (6.5–8 mm, average of four subjects). The green squares are from Holladay et al. (1991) (average of data with 4- and 5-mm pupil).

In accommodation, the eye's crystalline lens changes its power to minimize the blur of an image on the retina. When the distance to the object producing the image (the accommodative stimulus) is varied, the resulting response follows a pattern like the one in Figure 1A. For most stimulus distances, particularly near ones, the observed response is less than the stimulus (i.e., the eye appears to have focused to a farther distance than the stimulus); this is illustrated by the icon in the lower right of the figure. Such an error is called the *lag of accommodation*. At long distances, the response is nearer than the stimulus; this is illustrated by the icon in the upper left. This is the *lead of accommodation* (Morgan & Olmsted, 1939; Morgan, 1944, 1968; Heath, 1956; Fincham & Walton, 1957; Charman, 1999; Plainis et al., 2005). Lags of 1 diopter (D) or more have often been reported even for distances that are still within the range of distances to which the eye can change its state (i.e., distances farther than the near point and nearer than the far point). Stimulus–response curves like the one in Figure 1A have therefore become conventional wisdom in vision science, optometry, and ophthalmology (Ciuffreda, 2006; Chauhan & Charman, 1995). Our purpose here is to investigate whether accommodative errors—lags and leads—are as large as commonly thought.

Many hypotheses about the cause of the lags and leads have been offered. The most plausible ones fall into four categories that are not necessarily mutually exclusive.

### Depth of focus

In a perfect optical system, there is a surface where the image of an object is brought to sharp focus. Moving the object toward or away blurs the image on the surface. If the system had an infinitely sensitive blur detector, the distance through which an object could move before its image was judged to be out of focus would be infinitesimal. But the human eye is not a perfect optical instrument and the neural system is not infinitely sensitive to blur, so the range of object distances over which the image appears sharp is finite. This range is the visual system's *depth of focus*. The depth of focus depends on several factors, especially pupil diameter (Ogle & Schwartz, 1959; Green et al., 1980; Holladay et al., 1991), stimulus luminance (Tucker & Charman, 1986), and visual acuity (Heath, 1956; Green et al., 1980). Measured values for reasonably bright stimuli in people with normal acuity range from ±0.2–0.4D (Campbell, 1957; Ogle & Schwartz, 1959; Tucker & Charman, 1975, 1986; Sebastian et al., 2015).

Finite depth of focus should affect accommodative accuracy because small changes in stimulus distance would not affect perceived sharpness and therefore would not drive the system to change the power of the ciliary muscle. Thus, accommodative lags and leads may reflect a “dead zone” in which changes in distance are not detected (Bernal-Molina et al., 2014).

### Chromatic aberration

The human eye has different refractive powers for different wavelengths. Short wavelengths (e.g., blue) are refracted more than long (red), so blue and red images tend to be focused, respectively, in front of and behind the retina. The wavelength-dependent difference in refractive power is longitudinal chromatic aberration (LCA) (Marimont & Wandell, 1994; Thibos et al., 1992; Cholewiak et al., 2018). Ivanoff (1949) observed that with increasing accommodation (i.e., nearer and nearer focus), an ever-decreasing wavelength may be imaged sharply on the retina. That is, long wavelengths may be in focus on the retina when the stimulus is far and short wavelengths in focus when the stimulus is near. He proposed that the visual system utilizes LCA to “spare accommodation.” Specifically, the system accommodates only as much as needed to bring a span of wavelengths into focus. This idea is schematized in Figure 1B. The gray curve represents conventional lags and leads. The red, green, and blue lines represent wavelengths that, according to Ivanoff's hypothesis, would be in best focus at the retina when the stimulus is at different distances: red when the stimulus is far and blue when it is near.

There is good evidence that LCA is used to aid accommodative response (Kruger et al., 1993; Aggarwala et al., 1995; Cholewiak et al., 2017, 2018), but is it actually used to spare accommodation? Bobier et al. (1992) and Jaskulski et al. (2016) investigated this question. Bobier did so by manipulating the magnitude and sign of the eye's LCA by optical means. According to Ivanoff's hypothesis, increasing LCA magnitude should yield larger lags and leads (causing a decrease in the slope of the stimulus–response curve) while decreasing its magnitude should yield smaller lags and leads (causing an increase in slope). Bobier and colleagues observed no such effect: The slope of the stimulus–response curve did not change when they manipulated LCA magnitude. They concluded that lags and leads are not manifestations of a strategy to use different wavelengths for best focus at different stimulus distances. Jaskulski and colleagues tested the hypothesis by measuring accommodative responses to stimuli with narrow spectra (red, green, or blue) or a broad spectrum (white). If lags and leads are a by-product of focusing different wavelengths in a broad-spectrum light at different stimulus distances, one should observe steeper stimulus–response curves with narrowband than with broadband lights. Instead, they found no change in stimulus–response slope between narrow- and broadband stimuli.

Thus, there is no evidence to support the idea that accommodative lags and leads are a by-product of a strategy to use different wavelengths to focus at different distances and thereby spare accommodation.

### Control system

Control theory has been applied successfully to modeling biological systems, including accommodation. Figure 1C is a simplified diagram of a negative-feedback system for controlling accommodation (Toates, 1972; Stark et al., 1965; Schor, 1986; Kotulak & Schor, 1986b; Schor & Bharadwaj, 2006). The input is the image formed on the retina, which will be blurred if the eye is misaccommodated. Defocus error is created and serves as input to the controller in the middle of the diagram. The controller converts the error into a neural signal to drive the ciliary muscle and thereby change lens power. The controller has a “dead zone” around zero error where blur is not perceptible due to the eye's depth of focus (Campbell, 1957; Toates, 1972; Tucker & Charman, 1975). Within this range, no neural signal is generated. The falling and rising parts of the input–output curve represent errors that exceed the dead zone and drive the ciliary muscle's action in the correct direction to minimize defocus. The slopes of the falling and rising parts decrease at the extremes to yield the farthest and nearest distances to which the lens can adjust state: the far and near points. The change in accommodation creates a sharper retinal image, which is then fed back to the comparator to determine if the defocus has been sufficiently minimized.

Most control system models of accommodation assume proportional control to avoid overshooting and oscillation (Toates, 1972; Schor, 1986; Kotulak & Schor, 1986b; Schor & Bharadwaj, 2006). Specifically, a proportion less than 1 appears at the output so there will generally be an error present. Let i be the desired accommodative state, o the current state, e the error between desired and current (i–o), and g the gain of the proportional control. (For this simple development, we treat the controller as linear up to the near and far points, thereby ignoring the dead zone.) The output is related to the error as o = ge. If g is less than 1, only a fraction of the input appears at the output, so there will generally be an error present: That is, the output will not precisely equal the input even in steady state. This may be advantageous because the visual system's ability to sense a change in defocus is somewhat better when the eye is slightly out of focus than when it is perfectly focused (Campbell & Westheimer, 1958; Charman & Tucker, 1978). In other words, by maintaining an error, the system might be better able to respond rapidly to changes in stimulus distance.

### Objective vs. subjective measurement

Objective techniques (e.g., retinoscopy, autorefraction, wavefront aberrometry) are used widely to measure a patient's refractive error in order to prescribe an appropriate optical correction. But most clinicians fine-tune the prescription with a subjective test because the patient is often more satisfied with the correction indicated by that test (Strang et al., 1998). Many studies in which lags and leads of accommodation have been observed have used objective techniques, so it is worth considering whether the oft-reported accommodative errors are a consequence of the measurement technique.

Objective techniques use light reflected from the retina while subjective techniques use the visually relevant light absorbed by the photoreceptors. This inherent difference can cause differences in the measured state. Indeed, the refractive state measured objectively usually is more hyperopic than when measured subjectively (Freeman & Hodd, 1955; Glickstein & Millodot, 1970; Charman, 1975; Martin et al., 2011). There are many potential causes for the discrepancy.

1. Objective techniques analyze long-wavelength reflections. Those using visible light (e.g., retinoscopy) yield a reddish reflection. Those using infrared (autorefractors, wavefront sensors) yield infrared reflections. Subjective refractions are usually done with visible polychromatic light, so the most effective wavelength is shorter than those analyzed in objective techniques. Because of the eye's LCA, the shift toward longer wavelengths will make the eye appear more hyperopic with objective techniques (Llorente et al., 2003; Martin et al., 2011): That is, an apparent accommodative lag. One can of course account for the shift by using measurements of the eye's LCA (Marimont & Wandell, 1994).

2. The retinal layers responsible for the reflection are probably not the same as the layer responsible for subjective image quality. Anterior reflecting layers relative to the photoreceptive layer would cause a shift in the objective measurement toward hyperopia (i.e., an accommodative lag) (Glickstein & Millodot, 1970).

3. The retina is a thick reflector and different layers seem to have different directionality properties (Marcos et al., 1998). Some of the reflected light is guided by the photoreceptors toward the center of the pupil (Burns et al., 1995) while some is dominated by reflections from other sources and is directed more toward the pupil margins (Gao et al., 2009). For this reason, an eye may appear more myopic when measurements are weighted toward the pupil's margin rather than the center.

4. Differences in pupil size during objective and subjective measurements may cause differences in apparent refractive state. For example, most eyes have positive spherical aberration when focused at distance, meaning that marginal rays are focused anterior to paraxial rays (Porter et al., 2001; Salmon & van de Pol, 2006). Measurements with a large pupil may therefore indicate more myopia than measurements with a small pupil. In addition, the Stiles–Crawford effect (Stiles & Crawford, 1933), which decreases the effective size of the pupil (Bradley et al., 2014), affects subjective but not objective measurements. It is interesting to note that modeling and experiments indicate that subjective refractions (target that appears sharpest to the viewer) are relatively unaffected by changes in pupil size because such refractions are dominated by paraxial rays (Xu et al., 2013; Bradley et al., 2014). Objective measurements that give more weight to marginal rays may then be more affected by pupil size.

5. Higher-order aberrations could cause differences between objective and subjective measurements. The algorithm used by an objective technique in analyzing the reflected light may weight such aberrations differently than the subject's visual system does when performing a visual task. Some image-quality metrics applied to objective wavefront measurements have been able to predict subjective refraction reasonably accurately, which probably means that those metrics weight aberrations much like the visual system does (Martin et al., 2011; Thibos et al., 2004).

These objective–subjective differences will cause biases, mostly toward hyperopia (i.e., an accommodative lag). But they would also cause a lessening of the slope of the accommodation stimulus–response curve (Figure 1A) for the following reason. Spherical aberration is generally positive when the eye is accommodated far and shifts toward negative as the eye accommodates near (Cheng et al., 2004; Plainis et al., 2005). Others have pointed out that an objective algorithm that gives more weight to rays passing through the pupillary margin than the visual system does would then indicate an accommodative lead at far and a lag at near (Plainis et al., 2005; Buehren & Collins, 2006; Thibos et al., 2013), and this transition from an apparent lead to an apparent lag would cause a decrease in the slope of the stimulus–response curve.

### Consensus

The consensus view is that accommodative errors—lags and leads—are a by-product of the accommodative system changing state only as much as needed to bring an image into acceptable focus. The errors exist in part because of a “dead zone” where changes in response produce no perceptible change in image quality. Indeed, the errors may reflect a strategy of maintaining a state that is slightly off best focus because the accommodative system is then more sensitive to changes in stimulus distance than if it maintained focus perfectly (Campbell & Westheimer, 1958; Charman & Tucker, 1978; Bernal-Molina et al., 2014).

The consensus view is difficult to reconcile with two observations: (a) how visual acuity declines with small amounts of defocus and (b) the smallest change in stimulus distance that drives an accommodative response.

Several researchers have measured letter acuity as a function of the optical distance of the stimulus under well-controlled conditions (Tucker & Charman, 1975; Legge et al., 1987; Holladay et al., 1991; Guo et al., 2008; Legras et al., 2010; Zheleznyak et al., 2013). Accommodation was paralyzed and artificial pupils employed. Figure 1D shows that visual acuity was highest with no defocus and fell dramatically when the absolute value of defocus increased. Defocus of just 0.5D produced significant changes in acuity (over a factor of 2 in some of the studies). Why would the visual system tolerate accommodative errors of ∼1D (Figure 1A) that produce nontrivial changes in visual performance?

Kotulak and Schor (1986a) measured the smallest change in the optical distance of a target that elicits reliable accommodative responses. Stimulation was monocular with no change in target size at the retina. They observed consistent responses to 0.12D changes in distance. Why would the visual system tolerate errors as large as 1D when it can respond to much smaller changes?

### Experimental question

These observations motivated our experimental questions. At what distance is performance maximized when the eye attempts to accommodate to different distances? Specifically, are the distances at which visual acuity is best consistent with the oft-reported accommodative lags and leads? To answer these questions, we conducted subjective and objective measurements simultaneously. We emphasize the obvious point that the goal of accommodation should be to maximize visual performance and not to maximize some property of the image reflected from the retina. In other words, the most valid measure is subjective not objective.

## Method

### Participants

Six healthy adults (28.3 ± 5.6 years; three males) participated. Two were authors; the others were unaware of the experimental hypotheses. All had normal or corrected-to-normal visual acuity. Those requiring optical correction did so with contact lenses. Given their age, they are expected to have an accommodative range of ∼5.6D (Kasthurirangan & Glasser, 2006). Informed consent was obtained. Data from all of the recruited participants are included in this report. The research conformed to the tenets of the Declaration of Helsinki and was approved by the UC Berkeley Committee for Protection of Human Subjects.

### Hardware

In the main experiment, we utilized a novel display system with an integrated Shack–Hartmann wavefront sensor (FLIR Grasshopper GS3-U3-15S5M-C, FLIR systems, Wilsonville, OR, USA; coupled with a microlens array MLA150-7AR, Thorlabs Inc., Newton, NJ, USA), focus-adjustable lens (Optotune EL-10-30-TC; Optotune, Dietikon, Switzerland), and DLP projector (Texas Instruments LightCrafter 4710; Texas Instruments, Dallas, TX, USA) (Figure 3). The wavelength of the infrared light source for the wavefront sensor was 875 nm. The field of view was 12.5${}^{\circ }$ in diameter. An Optotune EL-10-30-TC focus-adjustable lens with a Comar 63 DN 25 Comar 63 DN 25 (Comar Optics, Linton, Cambridgeshire, UK) achromatic doublet offset lens was placed optically at the pupil-conjugate plane. As such, changes in the power of the adjustable lens did not cause changes in the magnification of the image at the eye. We used the adjustable lens to make fast (∼15 ms) changes in the optical distance to the stimulus. A model eye was used to confirm linear and stable defocus performance of the focus-adjustable lens from –1 to +6D. Stimuli were projected onto a screen by a Texas Instruments DLP LightCrafter 4500 with LED primaries and viewed by the subject's left eye. The spectra for the three primaries are provided in Supplementary Figure S8. Resolution was 62 pixels/deg for a Nyquist frequency of 31 cycles/deg. Stimuli were white and black; space-average luminance of the fixation stimulus was 138 cd/m${}^{2}$.

### Wavefront software

The Shack–Hartmann wavefront sensor was sampled at 70 Hz and videos were recorded for processing offline. For each video frame, wavefront spots were localized using robust subpixel template matching; pupil diameter was estimated from the observed spots using RANSAC. Outliers (spots that were malformed or too dim) were automatically filtered from further analysis. The spots were initially assumed to be 2D Gaussians for template matching. The templates were dynamically updated to account for changes in the spot spread due to individuals’ aberrations. Eye movements were discounted and corneal reflections well filtered via this method. Frames with too few spots or with noncircular pupils (e.g., due to blinks) were dropped. Zernike polynomials up to the sixth order (28 terms) were fit to the wavefront sensor data and ordered according to the Optical Society of America (OSA) standard (Thibos et al., 2002). Defocus was given by the coefficient of Zernike term $c_{2}^{0}$. We will refer to this as *RMS-based defocus* in the remainder of the article.

### Procedure

The key feature of the main experiment is that we simultaneously measured accommodation and visual acuity. Stimuli were presented to the left eye and wavefronts were measured on that eye as well. The right eye was patched. The apparatus (Figures 3A, B) enabled presentation of stimuli at various optical distances with no change in image size and simultaneous measurements of that eye's wavefront aberration and pupil diameter. On each trial, the subject first fixated a Maltese cross presented for 3 s at 0, 1, 2, 3, 4, 5, or 6D. These were the seven accommodative stimulus distances. The screen was then blanked for 150 ms, during which the power of the adjustable lens was changed to generate one of nine optical distances relative to the accommodative stimulus distance (−1.5, −1.0, −0.5, −0.25, 0, 0.25, 0.5, 1.0, or 1.5D). These are the relative stimulus distances. We measured visual acuity using a Tumbling-E letter acuity test. Letter size was 7.5minarc (Snellen equivalent of 20/30). The high-contrast letter was black on a white background. The spectrally broadband background enabled chromatic aberration to provide useful information for guiding accommodation (Kruger et al., 1993; Aggarwala et al., 1995; Cholewiak et al., 2018). The letter was presented in one of four orientations for 100 ms followed immediately by a 150-ms noise mask to prevent the subject from determining letter orientation from the after-image. Then a green Maltese cross was presented at the initial accommodative stimulus distance, and this signified that the subject should now indicate the letter's orientation in a four-alternative, forced-choice judgment. No feedback was provided. Once the response was recorded, the experiment proceeded to the next trial. This way, we presented stimuli at a variety of distances to stimulate accommodation and at the same time measured the distance at which the subject's visual acuity was greatest.

We note that our procedure is not the common procedure for measuring refractive state or accommodation in which a letter chart is presented and the subject is asked to accommodate to it. We instead use the letter E as a probe to find the distance relative to the accommodative stimulus at which acuity is highest.

We chose the Tumbling-E task for the subjective measurements because we wanted a demanding measure of visual performance and a measure that is familiar to subjects and practitioners. Letter acuity is a good choice because it is very sensitive to refractive error, retinal eccentricity, and many visual abnormalities (Herse & Bedell, 1989; Thorn & Schwartz, 1990; Levi & Klein, 1985) and is the “gold standard” for clinical assessment of spatial vision. (We note that two techniques for measuring accommodation—stigmatoscopy [Alpern & David, 1958] and laser optometry [Johnson, 1976; Owens, 1980]—are subjective in that they rely on a response from the subject. But neither involves complex pattern recognition like identifying a letter.) The letter presentations were too brief to cause an accommodative response (Figure 4A, “SI Appendix, Supplementary Figures S2–S6”) or a change in pupil diameter (“SI Appendix, Supplementary Figure S1”). By doing the objective and subjective measurements simultaneously, we were able to eliminate differences (pupil size, accommodative state) that might otherwise confound the comparison.

The experiment employed a randomized blocked design with trials blocked by the nine relative stimulus distances for each accommodative stimulus distance. There were 3,150 trials (7 accommodative distances × 9 relative distances × 50 repetitions) for each subject. Trials were distributed randomly between 10 sessions. Accommodation and pupil size were measured throughout with the wavefront sensor. At least 238 wavefront measurements were made on each trial.

### Analysis

From each subject, 700,000–1,000,000 wavefront measurements were collected. Each consisted of a time stamp, pupil size, and 28 Zernike coefficients. The measurements were made at 875 nm. We corrected them by 0.90D to account for the eye's LCA between the infrared source and the dominant wavelength of 555 nm in the stimuli (Marimont & Wandell, 1994). Objective accommodative responses to the fixation cross were estimated from the medians of the last 100 wavefront measurements of each stimulus presentation (∼1.5 s). Objective accommodative responses to the accommodative stimulus (letter E) were estimated from the median of the seven wavefront measurements captured during the presentation of the acuity stimulus (100 ms) (Figure 3C).

**Figure 2.**
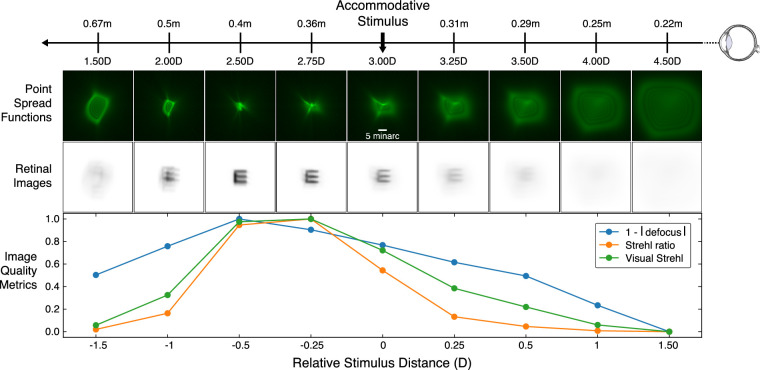
Effect of relative distance on retinal images and image-quality metrics. The accommodative stimulus is presented at 3.0D (0.33 m). A focus-adjustable lens changed the optical distance of the display (a relative change of 0, ±0.25, ±0.5, ±1.0, or ±1.5D) where the acuity target was presented. The first row indicates distances relative to the eye (not to scale). The second row shows point-spread functions (PSFs) for one subject. The PSFs were calculated from median Zernike fits for a 5-mm pupil at 550 nm. The third row shows associated retinal images for the letter E with a height of 7.5minarc (1.5minarc stroke width; 20/30 Snellen equivalent). The fourth row shows image-quality metrics computed from the wavefront measurements (Thibos et al., 2004). Blue is 1 − |defocus|, where defocus is RMS based, orange is Strehl ratio, and green is visual Strehl ratio. All metrics have been normalized from [0, 1] and are therefore unitless. Best image quality according to the metric is the peak value.

From each video frame, we used the Zernike coefficients to reconstruct the wavefront. A pupil function was calculated using the pupil size measured for that frame and was applied to the wavefront. The point-spread function (PSF) was then computed as the squared magnitude of the Fourier transform of the complex pupil function. Figure 2 shows PSFs from one subject when the accommodative stimulus was +3D. These PSFs are complex, so it is unclear what aspect of the set of PSFs would predict best perceived image quality and visual performance. We used four image-quality metrics: *RMS-based defocus*, *Seidel defocus*, *Strehl ratio*, and *visual Strehl ratio* (Thibos et al., 2004). Said another way, we used the wavefront data to generate four estimates of accommodation response distance (i.e., distance from the subject's eye, expressed in diopters).

**Figure 3.**
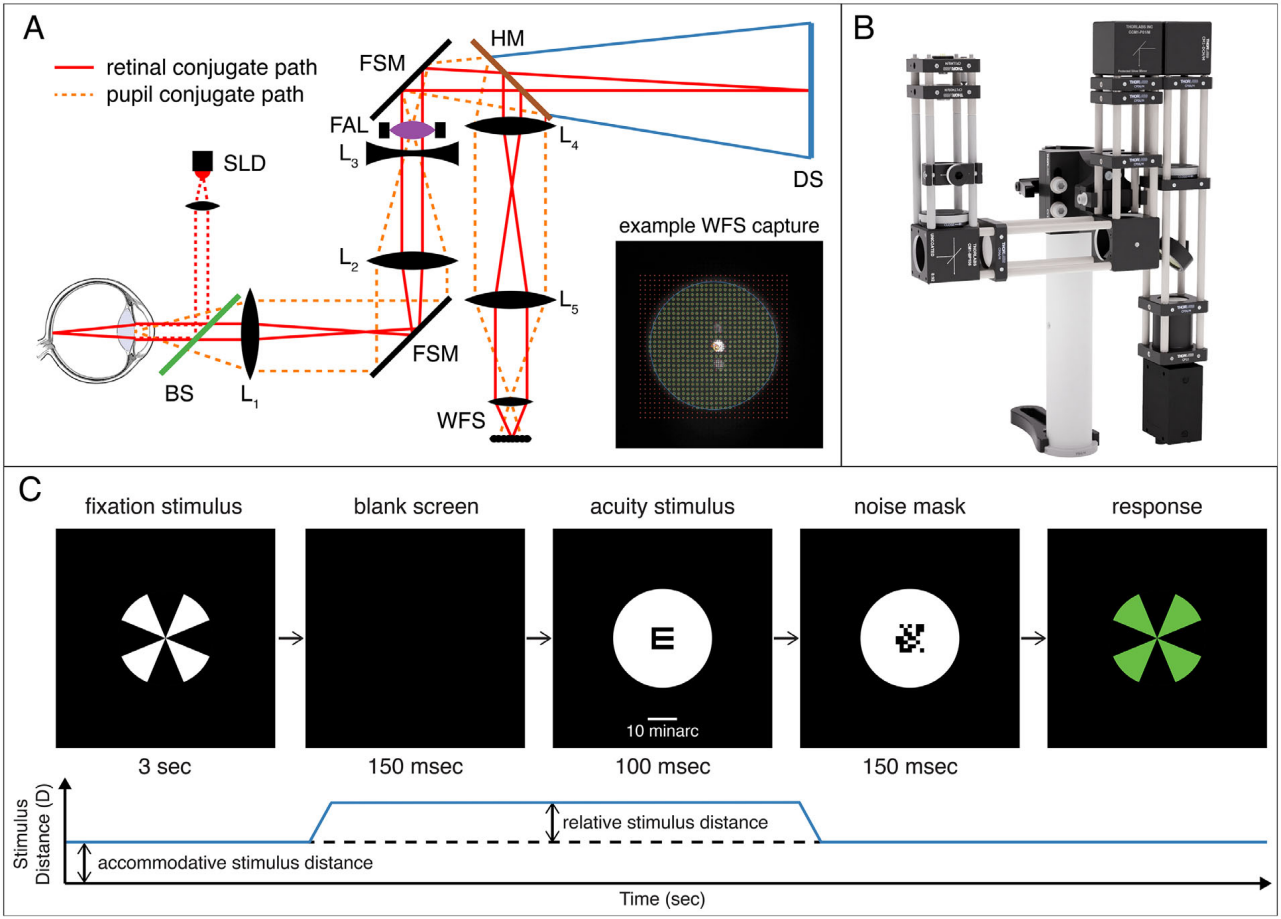
Experimental apparatus and method. (A) Schematic of the apparatus in the main experiment. Infrared light (875 nm) from the superluminescent diode (SLD) is collimated and reflects off a 5:95 R:T beamsplitter (BS) into the eye. The refracted wavefront is imaged via L${}_{1}$, front-surface mirror (FSM), and L${}_{2}$ to be conjugate with the focus-adjustable (FAL) and offset lens L${}_{3}$. The IR wavefront is then imaged onto the Shack–Hartmann wavefront sensor (WFS) via a hot mirror (HM), L${}_{4}$, and L${}_{5}$. The subject views the display screen (DS) through the system and hot mirror. The solid red lines illustrate the retinal-conjugate path and the dashed orange lines the pupil-conjugate path. Inset image is an example WFS capture with spots localized. (B) Rendered model of the display system. The subject's eye is located to the left and views a display screen to the right (not shown). (C) Experimental procedure for the main experiment with illustrated accommodative stimulus distance, provided by FAL, on bottom. Subjects initially fixate a Maltese cross at the accommodative stimulus distance for that trial. Then the screen is blanked and the optical distance of the screen is changed via the FAL to the desired relative stimulus distance for the acuity stimulus. The change in optical power took ∼15 ms (as shown by the blue line). An E in one of four orientations is briefly presented. Then the optical distance of the screen is returned to the accommodative stimulus distance while a dynamically changing noise mask is displayed to extinguish an afterimage of the E. A green Maltese cross is then shown, and this signifies that the subject should now indicate the perceived orientation of the E. Once the response is recorded, the white cross reappears and the next trial begins. The white bar in the middle panel indicates 10minarc.

RMS-based defocus is determined by fitting the aberrated wavefront with a spherical surface that minimizes RMS error. The response in diopters is

(1) $$R_{z}=\frac{c_{2}^{0}4\sqrt{3}}{r^{2}}$$

where $c_{2}^{0}$ is the Zernike defocus term that minimizes RMS and r is the radius of the pupil.

Seidel defocus is determined by fitting the aberrated wavefront with a spherical surface that matches the curvature of the two surfaces at the center of the pupil. The response in diopters is

(2) $$R_{s}=\frac{c_{2}^{0}4\sqrt{3}-c_{4}^{0}12\sqrt{5}+c_{6}^{0}24\sqrt{7}}{r^{2}}$$

where $c_{2}^{0}$, $c_{4}^{0}$, and $c_{6}^{0}$ are respectively the Zernike terms for defocus and primary and secondary spherical aberration.

Strehl ratio (SR) is

(3) $$SR=\frac{peak\left(PSF_{o}\left(x,y\right)\right)}{peak\left(PSF_{d}\left(x,y\right)\right)}$$

where $peak(PSF_{o})$ is the peak value of the measured PSF and $peak(PSF_{d})$ is the peak value of the diffraction-limited PSF. Strehl ratios approaching 1 indicate high image quality.

Visual Strehl ratio (VSX) is similar, but the observed and diffraction-limited PSFs are weighted by the inverse Fourier transform of the neural contrast sensitivity function:

(4) $$VSX=\frac{\int_{PSF}\left(PSF_{o}\left(x,y\right)N\left(x,y\right)dxdy\right)}{\int_{PSF}\left(PSF_{d}\left(x,y\right)N\left(x,y\right)dxdy\right)}$$

where N(x,y) is a neural weighting function.

**Figure 4.**
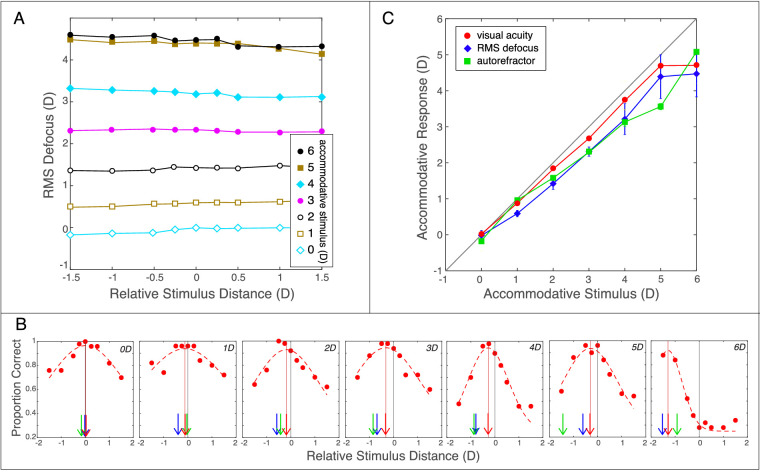
Accommodation during stimulus presentation, visual acuity, and stimulus–response curves for one subject. (A) Defocus as a function of relative stimulus distance for each accommodative stimulus. Data from the other subjects are quite similar (“SI Appendix, Supplementary Figures S2–S6”). For each accommodative stimulus distance, we found the defocus that minimized RMS error relative to the wavefront for each relative distance at which the letter E was presented (Equation 1). Defocus changed from ∼0D when the stimulus was 0D to ∼4D when the stimulus was 6D. Negative and positive values of relative stimulus distance represent distances respectively farther and nearer than the accommodative stimulus. There was no systematic change in defocus with relative distance, which means that the distance of the letter had no effect on the measured accommodative state. (B) Proportion correct in the visual acuity task for different accommodative stimulus and relative stimulus distances. Data from the other subjects are similar (“SI Appendix, Supplementary Figures S2–S6”). Each panel shows the data for one accommodative stimulus distance: from left to right 0, 1, 2, 3, 4, 5, and 6D. Within each panel, proportion correct in the acuity task is plotted as a function of the letter's distance relative to the accommodative stimulus. The vertical gray lines represent where letter distance was equal to accommodative stimulus distance. The red dashed curves are the best-fitting Gaussians (Equation 5). The vertical red lines and arrows represent the relative distance at which proportion correct was highest. Blue arrows indicate the relative distance at which RMS-based defocus was minimum and green arrows the relative distance indicated by the autorefractor. (C) Stimulus–response curves. Median accommodative response is plotted as a function of accommodative stimulus. Data from the other subjects are similar (“SI Appendix, Supplementary Figures S2–S6”). The gray line represents where response would precisely match the stimulus. The blue data are responses according to RMS defocus measured by the wavefront sensor. The green data are responses according to the autorefractor. The red data are responses according to best visual acuity. Error bars are standard deviations and are often smaller than the symbols. All of the error bars for best acuity are smaller than the symbols.

We also measured visual acuity at different distances relative to the accommodative stimulus distance. Proportion correct in the Tumbling-E acuity test was determined at each relative distance for every accommodative stimulus distance (Figure 4B). We fit the proportion-correct data with a Gaussian with the floor fixed at the chance rate of 0.25:

(5) $$g\left(d\right)=\left(a-0.25\right)e^{-\frac{1}{2}{\left(\frac{d-\mu }{\sigma }\right)}^{2}}+0.25$$

where d is the relative distance, a the maximum value, μ the mean, and σ the standard deviation. a, μ, and σ were free parameters. The distance associated with the maximum of the fit (μ) was the estimate of the relative distance that maximized visual acuity.

A bootstrap analysis was used to assess the variation in the accommodative responses estimated based on the subjective visual acuity task. Forty of the 50 repetitions were randomly sampled at each relative stimulus distance, and Equation 5 was fit to the sample to produce an estimate of the relative stimulus distance with peak visual performance. This sampling and fitting was repeated 1,000 times. The parameter means and standard deviations were calculated for the sampling distributions for each subject and accommodative stimulus distance.

### Autorefractor

We also measured accommodation with a commercial autorefractor (Grand Seiko WV-500, Grand Seiko, Tokyo, Japan; also called Shin-Nippon SRW-5000). The Grand Seiko WV-500 samples at approximately ∼1 Hz. Unfortunately, we could not measure visual acuity at the same time with this device due to physical constraints imposed by the autorefractor. The WV-500 projects bars arranged in a square pattern onto the retina and uses the separations of the bars in the reflected image to measure refractive error. For more detail on how it measures refractive state, see Mallen et al. (2001) and Wolffsohn et al. (2004). There are, of course, other commercial autorefractors (Pesudovs & Weisinger, 2004). They differ in the algorithms used to determine best-focus distance from the retinal reflection, so they may have revealed different results than our autorefractor findings.

The same subjects were tested in this experiment as in the main experiment, but as we mentioned above, we could not conduct the Tumbling-E acuity task in this experiment due to hardware constraints. The accommodative stimulus was the same Maltese cross. It was projected by the same DLP projector onto a screen at 1 m (1D) and viewed by the left eye. The right eye saw a dark uniform field. Accommodation was stimulated by placing ophthalmic lenses as close as possible to the left eye. Accommodation was measured in the right eye, which is appropriate because accommodation is yoked in the two eyes (Campbell, 1960; but see Vincent et al., 2015). Subjects’ refractive errors (including anisometropia) were corrected by contact lenses. Accommodative response was measured using the manual commercial mode (button click). The experimenter took three measurements at each accommodative stimulus distance for each subject. Medians and standard deviations of the responses are provided in “SI Appendix, Supplementary Figures S2–S6.” These data served as another objective measure of accommodative accuracy.

## Results

Figure 4 shows results from one of the subjects. (Individual data from the other subjects are provided in “SI Appendix, Supplementary Figures S2–S6.”) Figure 4A plots defocus as a function of the distance of the letter E relative to the accommodative stimulus. A relative distance of zero means that the letter was presented at the same distance as the accommodative stimulus. Negative and positive values correspond to letters presented respectively farther and nearer than the accommodative stimulus. The figure shows importantly that accommodative state did not vary as a function of where the letter appeared. In other words, this subject (and all the others; “SI Appendix, Supplementary Figures S2–S6”) held accommodative state constant during a critical portion of the trial. The figure also shows that the response distance (the distance at which defocus was minimized) varied systematically with accommodative stimulus distance.

Figure 4B plots the proportion of correct responses in the acuity task as a function of relative distance. Again, a relative distance of zero means that the letter appeared at the same distance as the accommodative stimulus. Performance was best when the relative distance was zero or slightly less than zero, except when the accommodative stimulus was at +6D, a distance to which this subject could not accurately accommodate because it was closer than her near point. We fit these data with Gaussians and used the relative distance associated with the peak of fitted curve as the subjective estimate of the accommodative response. Figure 4C plots the distance of the accommodative response as a function of the accommodative stimulus distance for three measures of the response. The diagonal line is where response distance would precisely equal stimulus distance: that is, no accommodative error. The blue and green data are the objective measurements: blue for the distances at which RMS defocus was minimum according to the wavefront sensor and green for the distances determined by the autorefractor. The red data are responses according to best visual acuity.

**Figure 5.**
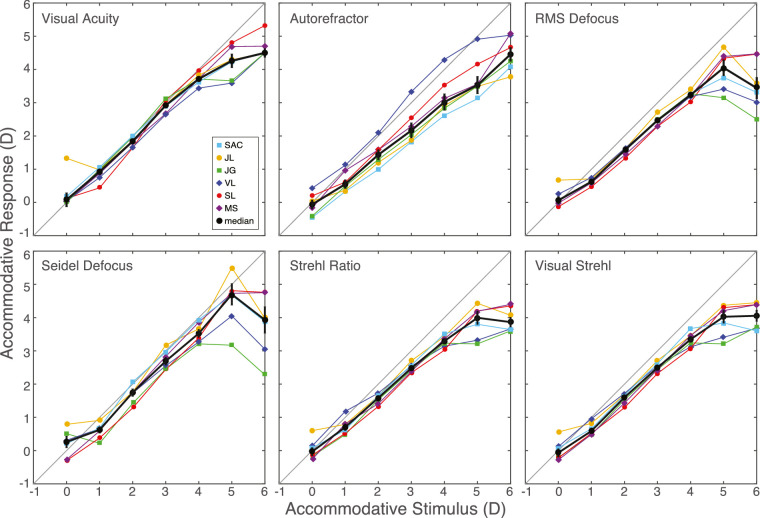
Accommodative response according to different metrics. Each panel plots accommodative response as a function of accommodative stimulus distance for one metric and for each subject. Black symbols represent the medians across subjects. The metrics in the upper row are respectively maximum of visual acuity, Grand Seiko WV-500 autorefractor, and RMS defocus. The metrics in the lower row are respectively Seidel defocus, Strehl ratio, and visual Strehl ratio. Error bars are standard errors.

The panels in the upper row of Figure 5 plot response distances as a function of the accommodative stimulus distance for the three measures of the response. The colored symbols and lines are the individual subject data. The black symbols and lines are the medians. Best-focus distance according to RMS defocus and the autorefractor exhibit typical accommodative lags: The eye appears to have not focused close enough to match the accommodative stimulus distance. The RMS defocus results indicate median lags of ∼1D for stimuli at 1–6D while the autorefractor results indicate lags of ∼0.5–1.5D over the same range. The red data are from the subjective measurements: the distances at which acuity was maximized. Those data exhibit little to no accommodative lag except at the nearest distances of 5 and 6D, which were nearer than the closest distance to which many of our subjects could accommodate; that is, 5 and 6D exceeded their near points.

We found therefore that accommodative errors are close to zero when the response is determined from visual performance. The median unsigned error across subjects and 0–4D stimulus distances were 0.15D (±0.08). Thus, the visual system accommodates sufficiently accurately to maximize performance in a visually demanding task. This means that commonly reported accommodative errors—lags and leads—are smaller than previously thought. We hasten to point out that our conditions are favorable for eliciting high visual performance: The stimulus has high contrast, fine detail, and high luminance, and our conditions are therefore favorable for eliciting accurate accommodation. We examine in the Discussion how accommodation is likely to be less accurate under less favorable conditions. It is interesting that we obtained very accurate accommodation even when some cues that are thought to aid accommodative accuracy—that is, target size and binocular disparity—were unavailable.

We next examined, as others have (Thibos et al., 2004; Martin et al., 2011; Thibos et al., 2013), whether some treatment of the objective wavefront data would yield results similar to the subjective measurements. We employed three common metrics: Seidel defocus (Equation 2), Strehl ratio (Equation 3), and visual Strehl ratio (Equation 4). For each subject and accommodative stimulus distance, we found the relative distance that maximized the Strehl and visual Strehl ratios. The panels in the lower row of Figure 5 plot the resulting data. Colored symbols and lines are individual subject data. Black symbols and lines are the medians. All three metrics indicate somewhat less accurate accommodative responses than the subjective measurements but more accurate than RMS defocus and the autorefractor results. Thus, some treatments of objective wavefront data appear to provide reasonable estimates.

**Figure 6.**
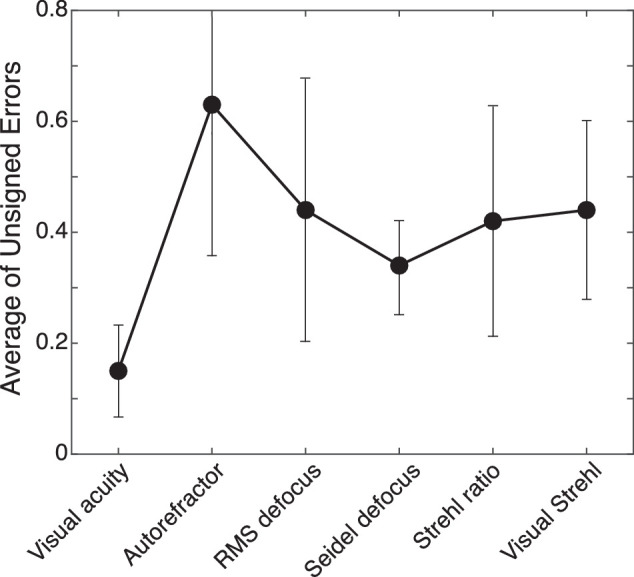
Accommodative errors according to different metrics. We calculated for each metric the median unsigned error across subjects for accommodative stimulus distances of 0, 1, 2, 3, and 4D. The data points are the averages across those distances for each metric. Error bars are standard deviations.

To compare the accuracy of the various means of measuring accommodative response, we computed, subject by subject, the unsigned error between response and stimulus. We did not include the data with accommodative stimulus distances of 5 and 6D because those distances were nearer than the near points of most of the subjects. The median errors across subjects and 0–4D stimulus distances are plotted in Figure 6. These data confirm the conclusion that visual acuity provided the most accurate and least variable response data and that Seidel defocus, Strehl ratio, and visual Strehl ratio provided reasonably accurate data from objective measurements. Those three metrics exhibited consistent lags of ∼1/3D, so one could in principle add 1/3D to bring them into better agreement with the subjective measurements.

## Discussion

### Previous work

Some previous work is superficially similar to ours but did not test at enough relative stimulus distances to determine where visual performance is maximized. Subbaram and Bullimore (2002) and Buehren and Collins (2006) measured accommodative responses to different stimulus distances and letter acuity at those same distances. They found that acuity was fairly constant across stimulus distances except for the nearest and farthest ones. Because they measured acuity at only the accommodative stimulus distances, one cannot determine from their data where visual performance is maximized. Johnson (1976) measured grating acuity as a function of the distance to the accommodative stimulus. He varied the luminance of the grating over a wide range. The grating was presented either at the accommodative stimulus distance or at the measured accommodative response distance. At low luminances, where accommodative lags and leads were large, Johnson observed an improvement in acuity when the grating was presented at the response distance rather than the stimulus distance. At high luminances, he observed a slight improvement in acuity when the grating was presented at the response distance. Because he only tested two distances relative to each accommodative stimulus distance, one cannot determine from Johnson's data the relative distance that maximizes visual performance.

### Subjective and objective refraction

The consensus view, based primarily on objective measurements, has been that accommodation exhibits substantial errors: leads at far distance and lags at mid to near distances. The lead-to-lag shift causes the slope of the stimulus–response curve to be less than 1. We now report that the lead and lag errors are quite small when measured subjectively such that the stimulus–response slope approaches 1.

Others have argued that spherical aberration is the primary source of differences between objective and subjective measurements (Plainis et al., 2005; Buehren & Collins, 2006; Tarrant et al., 2010; Thibos et al., 2013). They pointed out that most eyes exhibit positive spherical aberration when focused far and negative spherical aberration when focused near. We also observed this transition from positive to negative spherical aberration (“SI Appendix, Supplementary Figure S9”). Positive values mean that marginal rays are brought to focus anterior to paraxial rays: a lead due to marginal rays relative to paraxial rays. Negative values mean the opposite and cause an apparent lag. If conventional objective techniques such as autorefraction weighted marginal rays more than the visual system does, the objectively measured slope would be less than the subjectively measured one (Plainis et al., 2005; Buehren & Collins, 2006; Tarrant et al., 2010; Thibos et al., 2013; Xu et al., 2013; Bradley et al., 2014).

### Error signal for accommodation

The accommodative system needs an error signal to generate a response to minimize the error. There is good evidence that longitudinal chromatic aberration provides a useful signal (Kruger et al., 1993; Aggarwala et al., 1995; Cholewiak et al., 2018; Labhishetty et al., 2019) and some evidence that higher-order aberrations and microfluctuations of accommodation do as well (Fernández & Artal, 2005; Charman & Heron, 2015). Here we focus on changes in retinal-image contrast that can be used to guide accommodation. Our point is that there is sufficient information in contrast changes to support accommodation as accurate as we report here.

**Figure 7.**
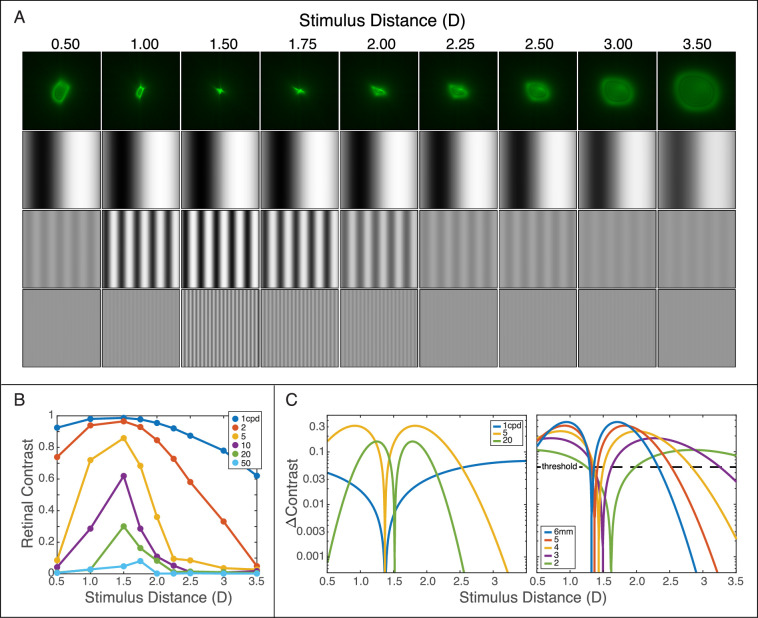
Stimulus distance, retinal contrast, and accommodative error signal. (A) Point-spread functions (PSFs) and retinal images of gratings. PSFs are plotted for one subject when the accommodative stimulus distance was 2D. Each panel represents the PSFs derived from the wavefront measurements at different distances relative to 2D. The associated retinal images are shown below for sinusoidal gratings at 1, 5, and 20 cpd. Object contrast was 1. They were obtained by convolving the PSFs with the gratings. (B) Retinal-image contrast for different spatial frequencies as a function of distance. Object contrast was 1. (C) Change in retinal contrast for ±0.125D changes in distance. The data in panel B were fit with Gaussians and the contrast changes calculated from those fits. The left panel shows retinal contrast changes for gratings of 1, 5, and 20 cpd and a 5-mm pupil. The right panel shows contrast changes for a 5-cpd grating and pupil sizes of 2–6 mm. The dashed line represents the contrast discrimination threshold for 5 cpd.

The top row in Figure 7A shows monochromatic PSFs for one subject when the accommodative stimulus was 2D. At distances farther and nearer relative to the best focus distance, 1.5D in this example, more defocus occurs so the PSFs spread. Below the PSFs, we show retinal contrasts at 1, 5, and 20 cpd for various distances. They are the convolution of the PSF with gratings of contrast 1. Defocus causes much more loss of contrast at 20 cpd than at 1 cpd (Green & Campbell, 1965; Charman & Tucker, 1977). Figure 7B plots retinal contrasts for the same conditions for a range of spatial frequencies. Again, the effect of a change in distance is much greater at high frequencies than at low. Also, the peak value shifts rightward (i.e., nearer) as spatial frequency increases, as has been observed before (Green & Campbell, 1965; Charman & Tucker, 1977).

We fit these data with Gaussians; they provided excellent fits. To create an error signal, we imposed ±0.125D changes in stimulus distance to determine how much change in retinal-image contrast would be caused by such changes in distance. The step size of ±0.125D corresponds approximately to the change in power the eye undergoes during accommodative microfluctuations (Charman & Heron, 2015) and to the smallest change that drives a consistent accommodative response (Kotulak & Schor, 1986a). The results are shown in Figure 7C: the left panel for different spatial frequencies with a fixed pupil diameter and the right panel for a fixed spatial frequency and different pupil diameters. The contrast-change signal increases from a small value near best focus to a peak value and then declines again at yet greater departures from the best-focus distance. The contrast change is greatest at 5 cpd and lower at 1 and 20 cpd. It is lower at 1 cpd because the slope of its through-focus contrast function is shallow (Figure 7B). It is lower at 20 cpd because retinal-image contrast is low even at best focus. The fact that the greatest change occurs at 5 cpd is consistent with the consensus view that spatial frequencies from 4–8 cpd provide the best signal for driving accommodation (Owens, 1980; MacKenzie et al., 2010; Burge & Geisler, 2011). Similarly, pupil diameter has a systematic effect, with larger diameters enabling larger contrast changes because of reduced depth of field. This is consistent with the observation that accommodation is most accurate when the pupil is large (Ward & Charman, 1985).

To drive an accommodative response, the neural visual system must be able to detect these contrast changes. To determine the change that should exceed threshold, we used contrast-discrimination functions at various spatial frequencies (Legge & Foley, 1980; Bradley & Ohzawa, 1986). In the right panel of Figure 7C, we show the just-noticeable change in contrast for a high-contrast grating at 5 cpd (Legge & Foley, 1980; Bradley & Ohzawa, 1986). Suprathreshold signals are generated at all pupil diameters, but much larger deviations in distance are required to exceed threshold with small pupils than with large ones. We next calculated the smallest change from the accommodative stimulus distance that produces a detectable change in contrast for an object contrast of 1. Lower contrast should not substantially affect the results, provided that the base contrast is suprathreshold, because the just-detectable change is roughly proportional to base contrast (i.e., the contrast-discrimination function nearly follows Weber's law). At near-threshold base contrasts, discrimination threshold rises significantly (Legge & Foley, 1980; Bradley & Ohzawa, 1986), so a comprehensive model would have to take that into account as well. The distances that should yield a discriminable change in contrast are provided in Table 1. The table shows that distance changes smaller than 0.2D provide a reliable signal to drive accommodation when the pupil is large (4–6 mm) at spatial frequencies of 5–20 cpd when contrast is high. These conditions, which are like the ones in our experiment, can promote accurate accommodation. As the pupil constricts or the image is blurred, the just-discriminable distances increase substantially, so these conditions should not promote accurate accommodation (Ward & Charman, 1985; Heath, 1956).

**Table 1.** Just-discriminable changes in distance. In the upper half of the table, pupil diameter is fixed at 5 mm and spatial frequency is varied. In the lower half, pupil diameter is varied and spatial frequency is fixed at 5 cpd. SF: spatial frequency in cpd. CDT: contrast-discrimination threshold. Pupil: pupil diameter in mm: ΔD: just-noticeable change in distance in diopters.

SF (cpd)	CDT	Δ D
1	0.070	∼ 4.0
2	0.059	0.56
5	0.045	0.09
10	0.043	0.07
20	0.060	0.13
50	0.100	∞

Pupil (mm)	CDT	Δ D

6	0.045	0.07
5	0.045	0.09
4	0.045	0.15
3	0.045	0.28
2	0.045	0.75

### Myopia development and accommodation

Accommodative responses of myopes are often different from those of emmetropes. Specifically, children, adolescents, and young adults with progressive myopia exhibit larger accommodative lags than age-matched emmetropes (Gwiazda et al., 1993; Abbott et al., 1998; He et al., 2005; Labhishetty & Bobier, 2017). In these studies, the myopic refractive error was corrected with spectacles or contact lenses and accommodation was measured objectively. Lags cause hyperopic defocus (image formed behind the retina), a situation that causes eye elongation (and hence myopia) in chickens, guinea pigs, tree shrews, and other animals (Wallman et al., 1978; Schaeffel & Feldkaemper, 2015). Thus, researchers and clinicians have hypothesized that the accommodative lags observed in young people with progressive myopia may be a stimulus for their eyes to lengthen and become myopic.

Our findings suggest that accommodative lags in young adults are smaller than indicated by objective measurements. Some, if not most, of the difference we observed is due to a greater contribution of spherical aberration to the objective measures than to subjective measurements. Positive spherical aberration can produce an apparent accommodative lead and negative spherical aberration an apparent lag (Plainis et al., 2005; Buehren & Collins, 2006; Tarrant et al., 2010; Thibos et al., 2013). Interestingly, young adult myopes exhibit more negative spherical aberration than emmetropes (Tarrant et al., 2010). If this is also the case in younger progressive myopes, the reported lags may be smaller than previously reported.

### AR/VR displays

Various stereoscopic displays, including augmented and virtual reality (AR and VR), create vergence-accommodation conflicts that can cause viewer discomfort and fatigue (Hoffman et al., 2008; Lambooij et al., 2009). Some AR/VR displays address this problem by incorporating adjustable optics to enable the optical distance of the screen to match the stereoscopic distance (and thereby the binocular vergence distance) of the object of interest (Koulieris et al., 2017; Padmanaban et al., 2017). But if accommodative lags and leads were really as large and variable as reported in the literature, adjusting the optical distance to match the vergence distance would produce accommodative errors as large as 1D relative to the object of interest (Figure 1A), and this would cause noticeable blur (Figure 1D). Our findings suggest that accommodation is actually quite accurate, so display engineers can achieve the best perceptual experience by equating optical and vergence distance.

## Supplementary Material

Supplement 1
