# Supplementary material for: Lags and leads of accommodation in humans: Fact or fiction?
Source: J Vis. 2021 Mar 25;21(3):21. doi: 10.1167/jov.21.3.21 (PMC7995353; doi:10.1167/jov.21.3.21)
Supplement: Supplement 1 [file jovi-21-3-21_s001.pdf]

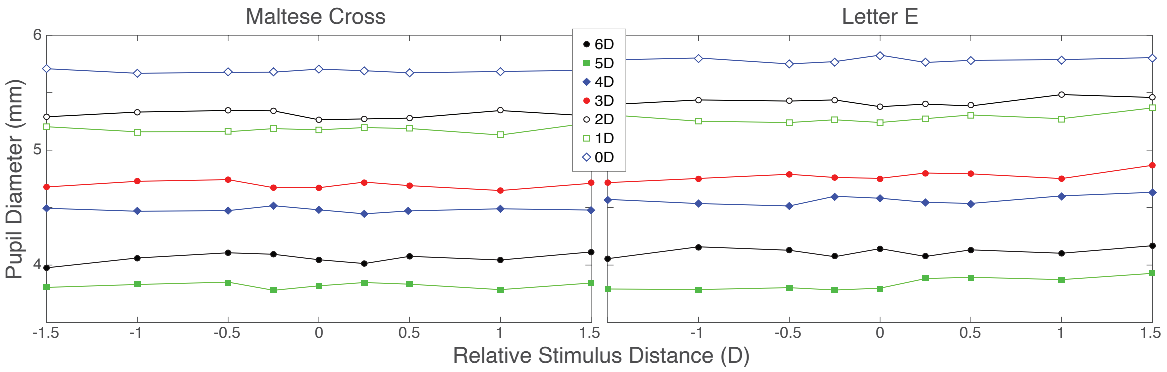

Figure S1: Pupil diameter during measurements of accommodation and visual acuity. Left panel: Pupil diameter during presentation of the Maltese cross for each of the seven accommodative stimulus distances and for each of the nine distances of the acuity target relative to the accommodative stimulus. The data have been averaged across subjects. Different colors represent different accommodative stimulus distances. The abscissa is the distance that the acuity target was presented relative to the accommodation stimulus. Zero means it was presented at the same distance. Right panel: Pupil diameter during the presentation of the letter E for each of the accommodative stimulus distances and for each of the distances of the acuity target relative to the accommodative stimulus. The data have again been averaged across subjects. Different colors again represent different accommodative stimulus distances. It is clear that pupil diameter did not change during the presentation of the acuity target relative to when fixating the Maltese cross.

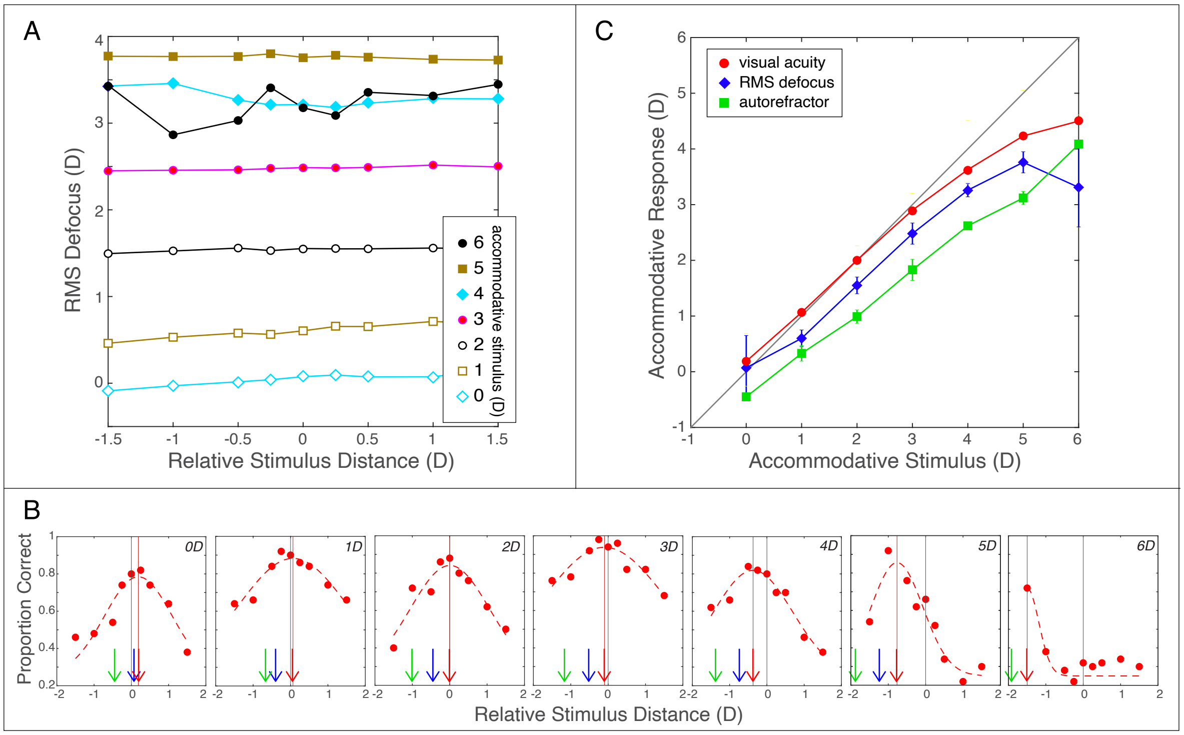

Figure S2: Data for subject SAC. Age: 35 years, myope. OD: –4.50DS; OS: –4.50DS where OD and OS refer to the left and right eyes, respectively, and DS refers to diopters of spherical correction. **A.** RMS defocus as a function of relative stimulus distance for the six accommodative stimulus distances (indicated in legend). Relative stimulus distance is the distance of the acuity stimulus relative to the distance of the accommodative stimulus. **B.** Proportion correct in the acuity task as a function of relative stimulus distance. Each panel shows the data from one of the six accommodative stimulus distances. The dashed red curves are Gaussian fits. Red vertical lines and arrows are the distances associated with the peak of the fitted Gaussians. Blue arrows indicate the defocus distances according to the wavefront sensor. Green arrows indicate the accommodative response distances according to the autorefractor. **C.** Accommodative response as a function of accommodative stimulus. Red indicates that distance at which visual acuity was maximized, blue the response distance according to RMS defocus as measured by the wavefront sensor, and green the response distance according to the autorefractor. Error bars indicate standard deviations. All of the error bars for best acuity are smaller than the symbols.

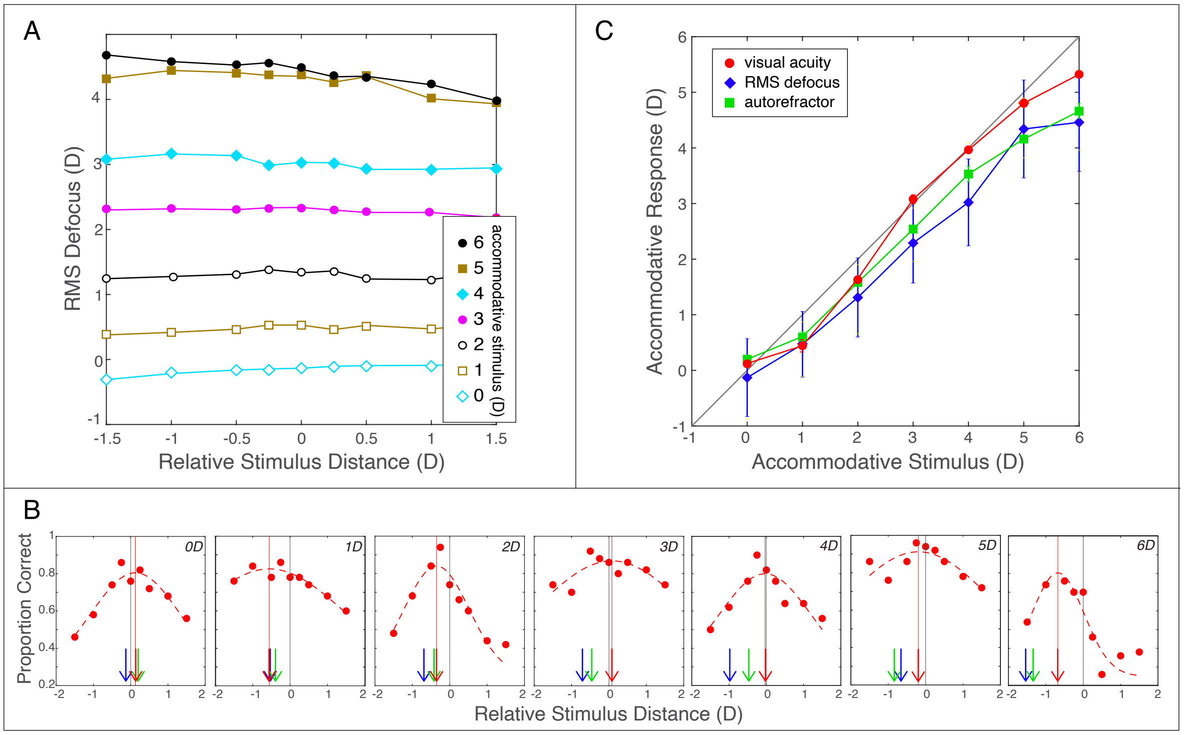

Figure S3: Data for subject SL. Age: 23 years, myope. OD: –5.75DS –3.25DC×2; OS: –4.50DS –3.50DC×180 where DS and DC refer to diopters of spherical and cylindrical correction, respectively. Error bars for best acuity are barely visible.

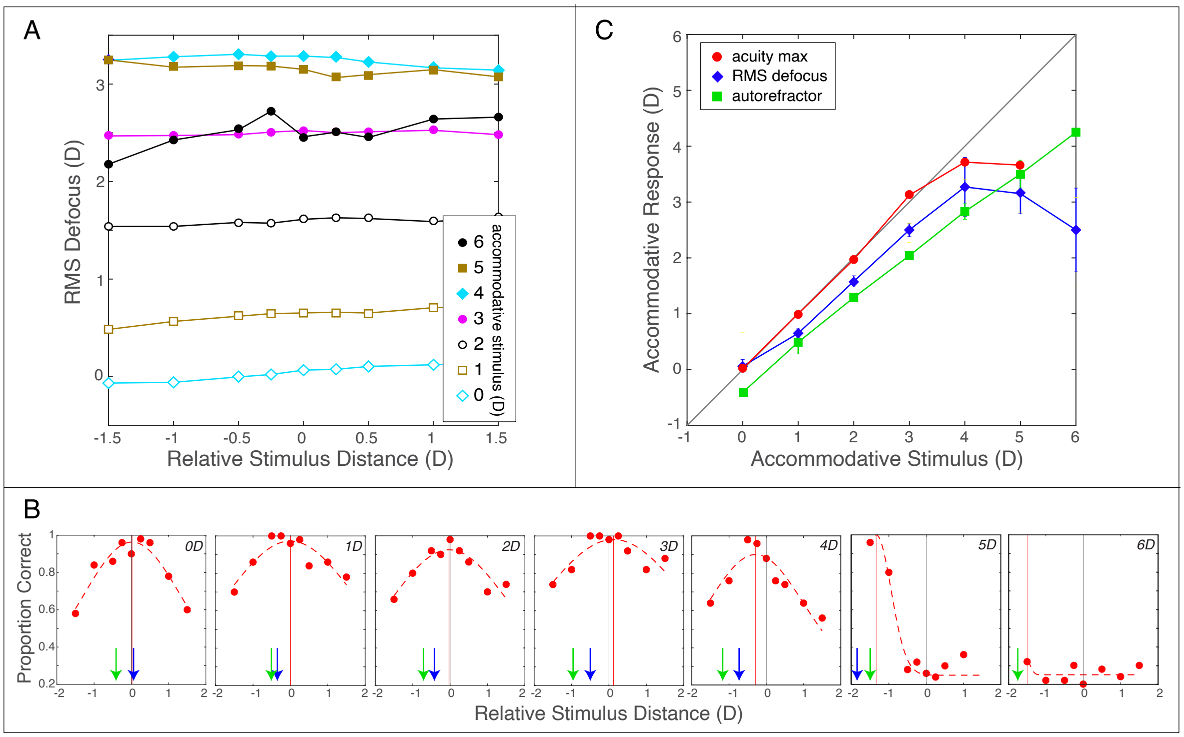

Figure S4: Data for subject JG. Age: 35 years, emmetrope in both eyes. Subject could not perform the acuity task reliably when the accommodative stimulus was +6D. Error bars for best acuity are smaller than the symbols.

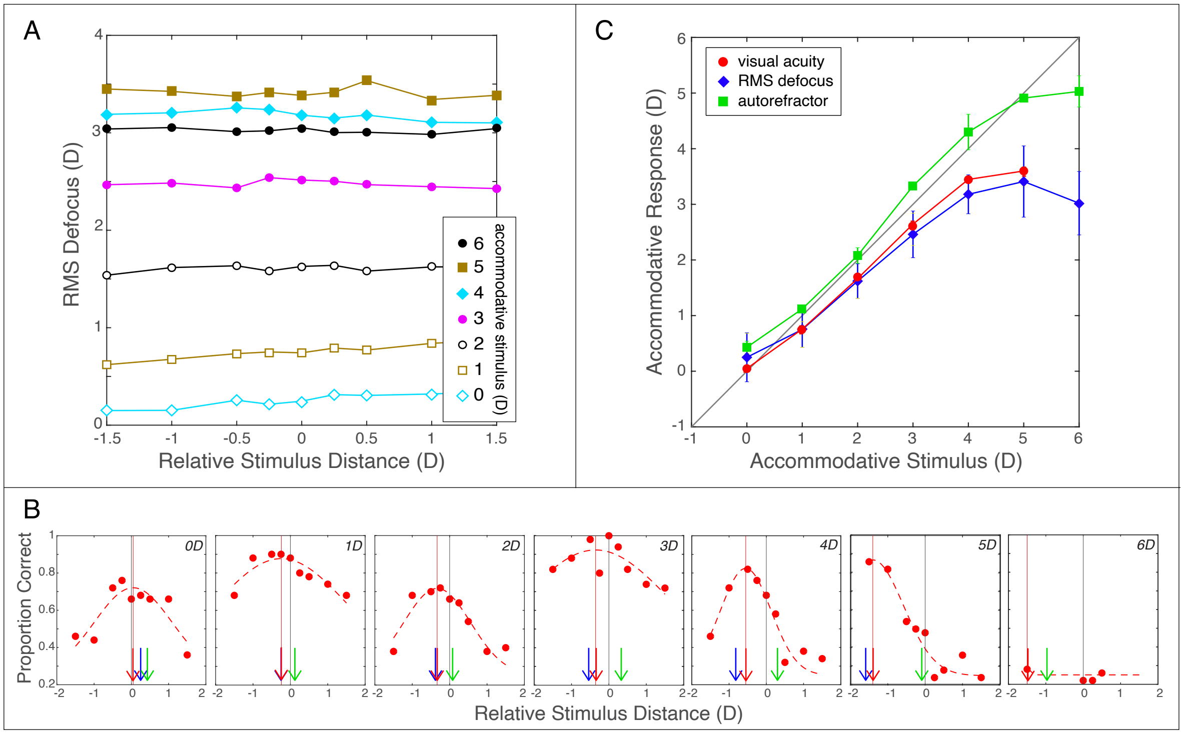

Figure S5: Data for subject VL. Age: 29 years, emmetrope in both eyes; myopic refractive error in both eyes corrected by Lasik surgery. Subject could not perform the acuity task reliably when the accommodative stimulus was +6D. Error bars for best acuity are barely visible.

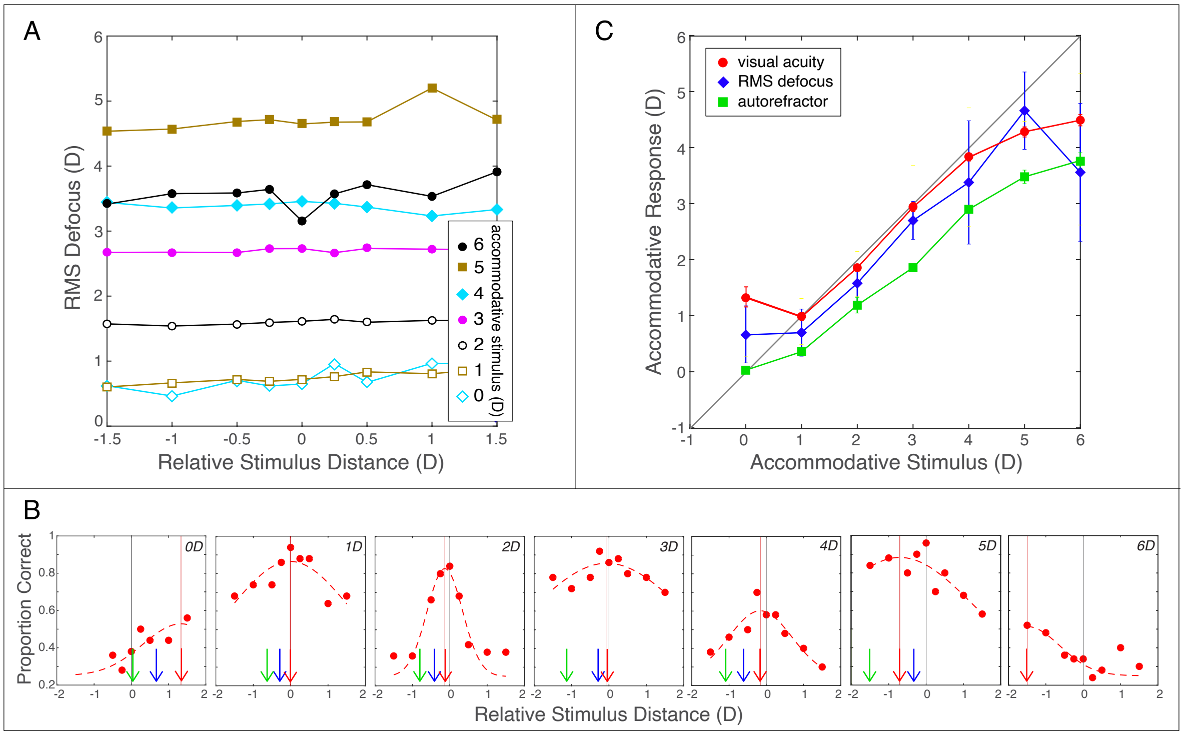

Figure S6: Data for subject JL. Age: 24 years, myope. OD: –3.50DS –1.25DC×10; OS: –3.50DS –2.25DC×165. Error bars for best acuity are barely visible.

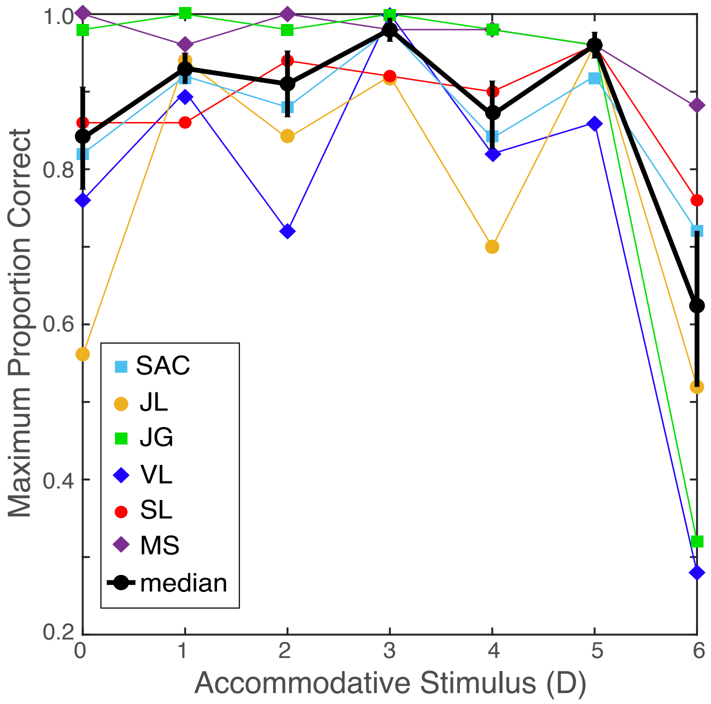

Figure S7: Maximum proportion correct in the acuity task at each accommodative stimulus distance. Colored symbols represent the values for each subject. Black symbols represent the medians. Error bars are standard are standard errors.

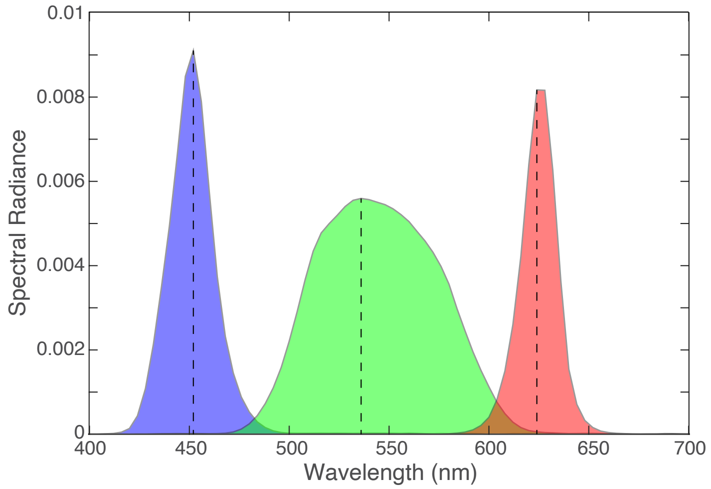

Figure S8: Radiance of the three primaries as a function of wavelength. Measurements were made with a Photo Research PR-650 SpectraScan Colorimeter from the position of the subject's eye. Dashed vertical lines indicate the peak radiance for each primary. The peak values are at 452, 536, and 624nm. The photopic luminances for the R, G, and B primaries at highest intensity were respectively 41.5, 221.8, and 8.5cd/m^2. The units of spectral radiance are $W \cdot sr^{-1} \cdot m^{-2} \cdot nm^{-1}$

.

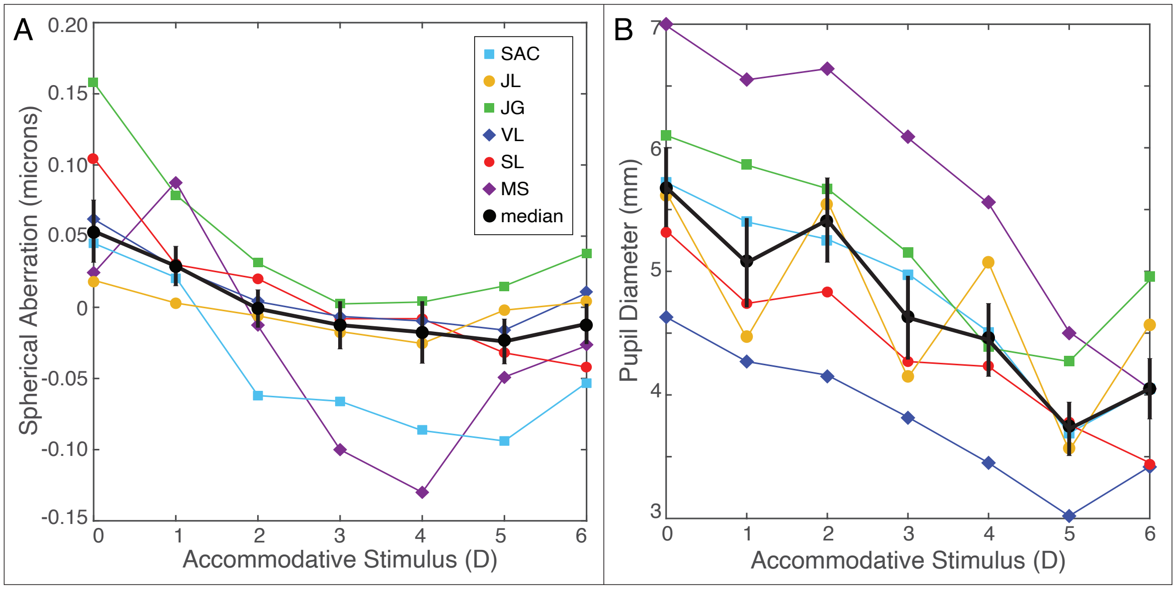

Figure S9: Primary spherical aberration (**A**) and pupil size (**B**) at each accommodative stimulus distance. Colored symbols represent median spherical aberration and pupil diameter as a function of accommodative stimulus distance for each subject. Black symbols represent the medians across subjects. Error bars are standard errors.
